# *Ozolaimus megatyphlon* and *Ozolaimus cirratus* parasitizing the *Iguana iguana* (Linnaeus, 1758) from Marajó Island, Pará, Brasil: new occurrence and morphological redescription

**DOI:** 10.1590/S1984-29612023046

**Published:** 2023-08-11

**Authors:** Vitória Luciana Paiva Canelas, Ricardo Luis Sousa Santana, Elaine Lopes de Carvalho, Elane Guerreiro Giese

**Affiliations:** 1 Universidade Federal Rural da Amazônia - UFRA, Belém, PA, Brasil; 2 Programa de Pós-graduação em Saúde e Produção Animal na Amazônia, Instituto da Saúde e Produção Animal, Universidade Federal Rural da Amazônia - UFRA, Belém, PA, Brasil; 3 Laboratório de Histologia e Embriologia Animal, Instituto da Saúde e Produção Animal, Universidade Federal Rural da Amazônia - UFRA, Belém, PA, Brasil

**Keywords:** Green iguana, parasites, nematodes, Ozolaimus, Pará, Iguana verde, parasitas, nematoides, Ozolaimus, Pará

## Abstract

This study aimed to redescribe two species of *Ozolaimus*, parasites of free-living green iguanas native to Marajó Island. The gastrointestinal system of four iguana specimens was evaluated for the presence of helminths. Altogether, 12,028 nematodes were found, with a prevalence of 100%, an infection range of 780 to 7,736 nematodes, an infection intensity of 3.007, and a mean abundance of 3,007. Light microscopy and scanning electron microscopy were used to determine the species of nematodes found. The cecum was the site of infection that had the highest parasitic load. Morphologically, the nematodes were compatible with the genus *Ozolaimus* Dujardin, 1844, with the species *Ozolaimus megatyphlon* (Rudolphi, 1819) Dujardin, 1845, and *Ozolaimus cirratus* Linstow, 1906. Scanning electron microscopy showed the presence of small structures (serrated in *Ozolaimus cirratus* and rounded in *Ozolaimus megatyphlon*) located below the esophageal leaves. We also evidenced the phasmids in both species; this is the first record of these structures in nematodes of the genus *Ozolaimus*. In addition, this work expands the records on the geographic distribution of these parasites.

## Resumo

Este estudo tem como objetivo redescrever duas espécies de *Ozolaimus*, parasitas de iguanas verdes de vida livre nativas da Ilha de Marajó. O sistema gastrointestinal de quatro espécimes de iguana foi avaliado quanto à presença de helmintos. Ao todo, foram encontrados 12.028 nematoides, com prevalência de 100%, intervalo de infecção de 780 a 7.736 nematoides, intensidade de infecção de 3.007 e abundância média de 3.007. Microscopia de luz e microscopia eletrônica de varredura foram utilizadas para determinar as espécies de nematoides encontradas. O ceco foi o local de infecção que apresentou maior carga parasitária. Morfologicamente, os nematoides eram compatíveis com o gênero *Ozolaimus* Dujardin, 1844, com as espécies *Ozolaimus megatyphlon* (Rudolphi, 1819) Dujardin, 1845 e *Ozolaimus cirratus* Linstow, 1906. A microscopia eletrônica de varredura mostrou a presença de pequenas estruturas (serrilhadas em *Ozolaimus cirratus* e arredondadas em *Ozolaimus megatyphlon*) localizado abaixo das folhas esofágicas. Também foram evidenciados os fasmídeos em ambas as espécies; este é o primeiro registro dessas estruturas em nematoides do gênero *Ozolaimus.* Além disso, este trabalho amplia os registros sobre a distribuição geográfica desses parasitos.

## Introduction

*Iguana iguana* (Linnaeus, 1758), popularly known as the green iguana, belongs to the order Squamata (Lepidosauria: Reptilia), the most diversified among the group and one of the most important, which includes about 19 families and 4,500 species of lizards (Barten, 2006; Vitt & Caldwell, 2008). *Iguana iguana* is distributed throughout the Americas, including Brazil and occurs in the Amazon, Caatinga and Pantanal biomes (Campos & Desbiez, 2013).

Due to their low cost and ready availability, green iguanas have become one of the most popular unconventional pets in Brazil, making them economically important (Bauer & Bauer, 2014). Additionally, they are herbivorous, which many owners prefer, as they do not have to deal with providing insects or rodents for food (Barten, 1993).

These reptiles are hosts for a wide variety of parasites, which can be acquired by ingestion of contaminated plant material, coprophagy, geophagy, or active penetration by nematode larvae (Anderson et al., 2009). The authors Loukopoulos et al. (2007) and Breves et al. (2011) demonstrated that helminths of the Oxyurida order are commonly found parasitizing the gastrointestinal system of green iguanas.

Despite the growing number of studies related to host parasites of green iguanas in Brazil, most studies are concentrated in the Southeast, Midwest, and Northeast regions of the country, leaving gaps regarding helminth fauna in the northern region. The Amazon region still lacks information on helminths related to lizards. With that in mind, this study aimed to redescribe two species of the genus *Ozolaimus* of *Iguana iguana* from Marajó Island in the State of Pará.

## Material and Methods

From 2019 to 2021, four free-living specimens of *I. iguana* were acquired dead from residents of the municipality of Soure (00º 43' 00” S; 48º 31' 24” W), Marajó Island, State of Pará. The research was carried out under authorization from Sisbio nº 68028. The organs of the digestive system, such as the esophagus, stomach, small intestine, and large intestine (colon, cecum, and rectum), were transported refrigerated to the laboratory. In the laboratory, each organ was isolated in plastic trays containing 0.9% NaCl saline and analyzed under a Leica ES2 stereomicroscope (Leica Microsystems GmbH, Wetzlar, Germany) to investigate the presence of helminths.

The collected nematodes were washed in 0.9% NaCl physiological solution, fixed in AFA solution (93 parts of 70% ethyl alcohol, 5 parts of formaldehyde, and 2 parts of glacial acetic acid), and stored in 70% alcohol. For light microscopy (LM), the nematodes were clarified in a 30% Lactophenol Aman solution and photographed in a Leica DM2500 microscope with a DFC310 FX digital capture system with Leica Application Suite V4.4 software (Leica Microsystems GmbH, Wetzlar, Germany). They were drawn and measured using a Leica DM2500 microscope with an imaging tube attached. The drawings were measured with the aid of a ruler, and the measurements were converted to micrometers or millimeters according to the Leica DM2500 (Leica Microsystems GmbH, Wetzlar, Germany) measurement conversion table. For morphometric analysis, 25 males and 20 females were used. After those procedures, the nematodes were stored in glycerin alcohol (70% ethanol with 5% glycerin). Measurements are given in millimeters, unless otherwise noted, and are presented as average values followed by minimum and maximum values in parentheses.

For scanning electron microscopy (SEM), the nematodes fixed in AFA solution were washed with distilled water, post-fixed in 1% osmium tetroxide for 2 hours, and then submitted to dehydration in an increasing series of ethanol from 70% ethanol until 100% for 1 hour in each battery of alcohol, subsequently subjected to the critical point of CO_2_ model K850 Critical Point Dryer (Quorum Technologies Ltd., England), mounted on metallic aluminum supports (stubs), metallized with gold+palladium, and analyzed in a scanning electron microscope model VEGA 3 LMU (TESCAN, Brno, Czech Republic). Scientific articles and dichotomous keys were used to identify the species: Dosse (1942), Leussink (1958), Vicente et al. (1993), Anderson et al. (2009), and Gibbons (2010).

Voucher specimens for the parasites were deposited in the Coleção Helmintológica do Instituto Oswaldo Cruz (CHIOC), Manguinhos, Rio de Janeiro, Brazil, as: CHIOC 39620 a-h for males and females of *O*. *cirratus* and CHIOC 39621 a-h for males and females of *O*. *megatyphlon*, correspondingly.

## Results

A total of 12,028 nematodes were recovered from four specimens of *I. iguana*, with prevalence of 100% (n=4), mean intensity of infection of 3,007, mean abundance of 3,007, and range of infection of 780 to 7,736 nematodes. The parasites were found (mixed infection) in the small and large intestine (colon, cecum, and rectum). The cecum was the site with the highest rate of infection. The specimens collected are morphologically compatible with the genus *Ozolaimus* Dujardin, 1844. In our study, we identified the species *Ozolaimus megatyphlon* (Rudolphi, 1819), Dujardin, 1845, and *Ozolaimus cirratus*, and their morphometries were compared to others already described in the literature for these two species (Tables 1 and 2).

Order Oxyurida,

Family Pharyngodonidae Travassos, 1920

Genus *Ozolaimus* Dujardin, 1844

Specie *Ozolaimus megatyphlon* (Rudolphi, 1819) Dujardin, 1845

*Ozolaimus cirratus* Linstow, 1906

**Table 1:** Comparison of morphometric data of specimens of *Ozolaimus megatyphlon* from *Iguana iguana* on Marajó Island, PA, with data from other authors. a: The front-end, b: µm, #: number.

	***Ozolaimus megatyphlon***		***O*. *megatyphlon***		***O*. *megatyphlon***		***O*. *megatyphlon***	
**Host**	***Iguana iguana***		*Iguana iguana rhinolopha*		*Iguana iguana*		*Iguana iguana*	
**Location**	**Marajó, Pará, Brazil**		Mexico		Alagoas, Maranhão, Goiás and Mato Grosso, Brazil		Maranhão, Brazil	
**Habitat**	**Cecum e intestine**		Cecum		Cecum, colon and rectum		Large intestine	
**References**	**Present study**		Caballero, 1938		Breves et al., 2011		Otávio et al., 2018	
	**Female**	**Male**	Female	Male	Female	Male	Female	Male
**Total body length**	**4.80-7.57**	**4.71-6.28**	6.25-7.40	4.85-5.90	4.99-6.97	3.67-4.76	7.10-8.10	5.40-5.50
**Body width**	**0.13-0.80**	**0.33-0.61**	0.62-0.72	0.39	0.56-0.85	0.34-0.40	0.70-0.80	0.50-0.70
**Nerve Ring^a^**	**0.22-0.37**	**0.10-0.80**	-	-	0.30-0.80	0.20-0.30	-	-
**Excretory pore**	**1.30-2.74**	**1.08-2.14**	-	-	-	-	2.40-2.80	1.80-2.00
**1a - Length of the 1º part of the esophagus**	**0.74-1.20**	**0.50-0.86**	-	-	1.04-1.16	0.66-0.96	1.15-1.36	0.90-0.95
**1b - Width of 1st part of the esophagus**	**0.07-0.24**	**0.10-0.16**	-	-	-	-	-	-
**2a - Length 2nd part of the esophagus**	**0.42-1.37**	**0.30-1.02**	-	-	1.39-1.71	0.89-1.22	1.17-1.62	1.10-1.16
**2b - Width of the 2nd portion of the esophagus**	**0.05-0.19**	**0.04-0.53**	-	-	-	-	-	-
**Basal bulb length**	**0.17-0.36**	**0.14-0.28**	-	-	0.18-0.25	0.10-0.19	0.21-0.23	0.22
**Basal bulb width**	**0.13-0.29**	**0.14-0.22**	-	-	0.18-0.28	0.16-0.20	0.25-0.28	0.20-0.23
**Total esophagus**	**1.56-2.77**	**1.20-1.90**	-	-	2.43-2.87	1.68-2.09	2.45-2.90	2.00-2.10
**Spicule**	**-**	**0.80-2.10**	-	1.07-1.18	-	0.72-1.03	-	1.01-1.20
**Tail^b^**	**200-510**	**70-200**	-	-	-	-	-	-
**Distance from vulva to anus**	**1.00-1.94**	**-**	-	-	-	-	1.57-1.80	-
**Distance from vulva to end of tail**	**1.03-2.50**	**-**	1.35-2.65	-	0.98-1.58	-	-	-
**Length of eggs^b^**	**100-980**	**-**	130-140	-	100-150	-	100-120	-
**Egg width^b^**	**50-80**	**-**	69-70	-	60-90	-	60-70	-
**# specimes**	**20**	**25**	-	-	-	-	10	10

**Table 2:** Comparison of morphometric data of specimens of *Ozolaimus cirratus* from *Iguana iguana* from Marajó Island, with data from other authors. a: The front-end, b: µm, #: number.

	**Ozolaimus cirratus**		**O. cirratus**		**O. cirratus**		**O. cirratus**		**O. cirratus**	
**Host**	***Iguana iguana***		*Iguana tuberculata*		*Iguana tuberculata*		*Iguana iguana*		*Iguana iguana*	
**Location**	**Marajó, Pará, Brazil**		Alemanha		México		Alagoas, Maranhão, Goiás and Mato Grosso, Brazil		Maranhão, Brazil	
**Habitat**	**Large intestine, Ceca**		Intestine		?		cecum and colon		Small intestine	
**References**	**Present study**		Linstow, 1906		Dosse, 1942		Breves et al., 2011		Otávio et al., 2018	
	**Female**	**Male**	Female	Male	Female	Male	Female	Male	Female	Male
**Total body length**	**4.46-7.77**	**5.00-7.28**	6.20	5.66	8.26-10.95	5.17-6.52	6.38-8.73	4.90-6.54	7.70-8.00	5.75-6.90
**Body width**	**0.53-0.86**	**0.40-0.63**	0.79	0.53	0.75-1.00	0.50-0.58	0.84-1.10	0.57-0.60	0.80-1.00	0.60-0.67
**Nerve Ring^a^**	**0.29-0.37**	**0.17-0.29**	-	-	0.20-0.31	0.25-0.38	0.40-0.60	0.20-0.30	-	-
**Excretory pore**	**1.66-3.14**	**1.19-2.01**	-	-	2.00-3.57	1.50-2.29	-	-	2.60-2.80	2.32-2.50
**1a - Length of the 1º part of the esophagus**	**0.71-1.22**	**0.52-0.74**	-	-	0.78-1.06	0.58-0.83	1.05-1.16	0.74-0.91	1.10-1.15	0.81-0.91
**1b - Width of 1st part of the esophagus**	**0.13-0.31**	**0.14-0.20**	-	-	-	-	-	-	-	-
**2a - Length 2nd part of the esophagus**	**0.50-1.00**	**0.36-0.72**	-	-	0.90-1.33	0.73-1.00	1.19-1.36	0.89-1.13	1.05-1.17	0.77-1.02
**2b - Width of the 2nd portion of the esophagus**	**0.04-0.17**	**0.06-0.13**	-	-	-	-	-	-	-	-
**Basal bulb length**	**0.17-0.38**	**0.14-0.27**	-	-	0.11-0.23	0.15-0.20	0.29-0.37	0.20-0.27	0.30-0.35	0.18-0.28
**Basal bulb width**	**0.17-0.26**	**0.11-0.20**	-	-	-	-	0.27-0.34	0.21-0.25	0.25-0.30	0.24-0.27
**Total esophagus**	**1.55-2.80**	**1.12-2.15**	-	-	-	-	2.25-2.45	1.65-2.04	2.20-2.30	1.70-1.92
**Spicule**	**-**	**1.50-2.37**	-	2.20	-	1.80-2.10	-	2.08-2.37	-	1.80-2.50
**Tail^b^**	**170-510**	**110-230**	-	-	300-480	110-180	-	-	-	-
**Distance from vulva to anus**	**1.05-1.80**	**-**	-	-	-	-	-	-	1.98-2.80	-
**Distance from vulva to end of tail**	**1.42-2.22**	**-**	-	-	2.00-3.47	-	1.40-3.03	-	-	
**Length of eggs^b^**	**80-120**	**-**	90	-	140-150	-	120-140	-	110-130	-
**Egg width^b^**	**50-70**	**-**	60	-	60-70	-	60-100	-	60	-
**# specimes**	**20**	**25**	-	-	20	10	-	-	10	10

### *Ozolaimus megatyphlon* (based on light microscopy and scanning electron microscopy. Figures 1 [Fig gf02] [Fig gf03] to 4; Table 1)

**Figure 1:**
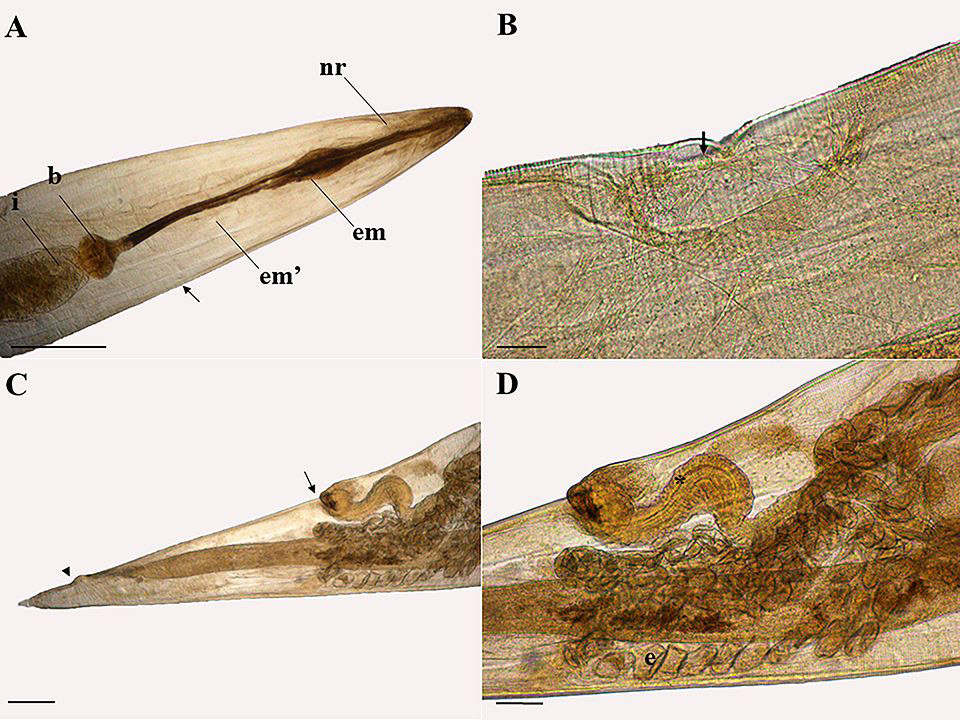
Light microscopy of female *Ozolaimus megatyphlon,* a parasite of *Iguana iguana*. A. Anterior extremity, nervous ring (nr), esophagus first portion (em), and muscular esophagus second portion (em'), prebulbar excretory pore (arrow), bulb (b), intestine (i). Bar=300µm. B. Excretory pore (arrow). Bar=60µm. C. Posterior end, lateral view, the vulva (arrow) and anal opening (arrowhead). Bar=300µm. D. Muscular vagina (*), uterus with eggs (e). Bar=100µm.

**Figure 2 gf02:**
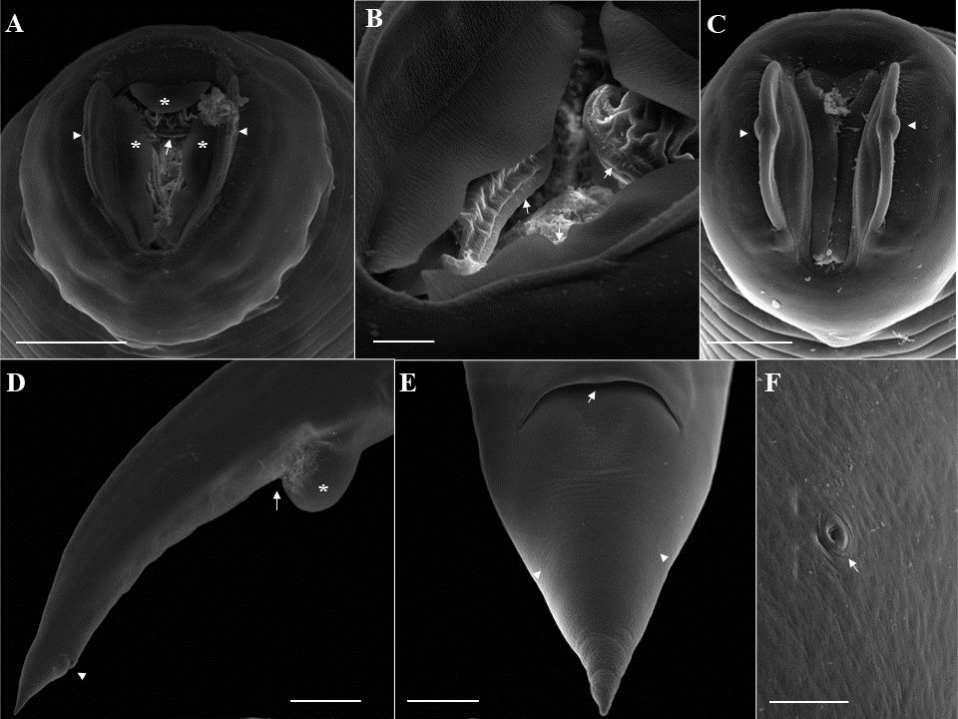
Scanning electron microscopy of female *Ozolaimus megatyphlon,* a parasite of *Iguana iguana*. A. Anterior end, projection of 3 tripartite cuticular membranes from the esophageal segment (*). Orifice of the amphids canal (arrowhead) and esophageal leaves (arrow). Bar=50µm. B. Esophageal leaves with rounded structure (arrow). Bar= 10µm. C. Anterior extremity, slit mouth, and presence of lips with lateral cephalic papilla (arrowhead). Bar=20µm. D. Posterior end, lateral view, vulva (arrow) whose anterior lip is projected (*), and anal opening (arrowhead). Bar=200µm. E. Posterior end, ventral view, tail, anal opening (arrow), and phasmids (arrowhead). Bar=50µm. F. Phasmid (arrow). Bar=5µm.

**Figure 3 gf03:**
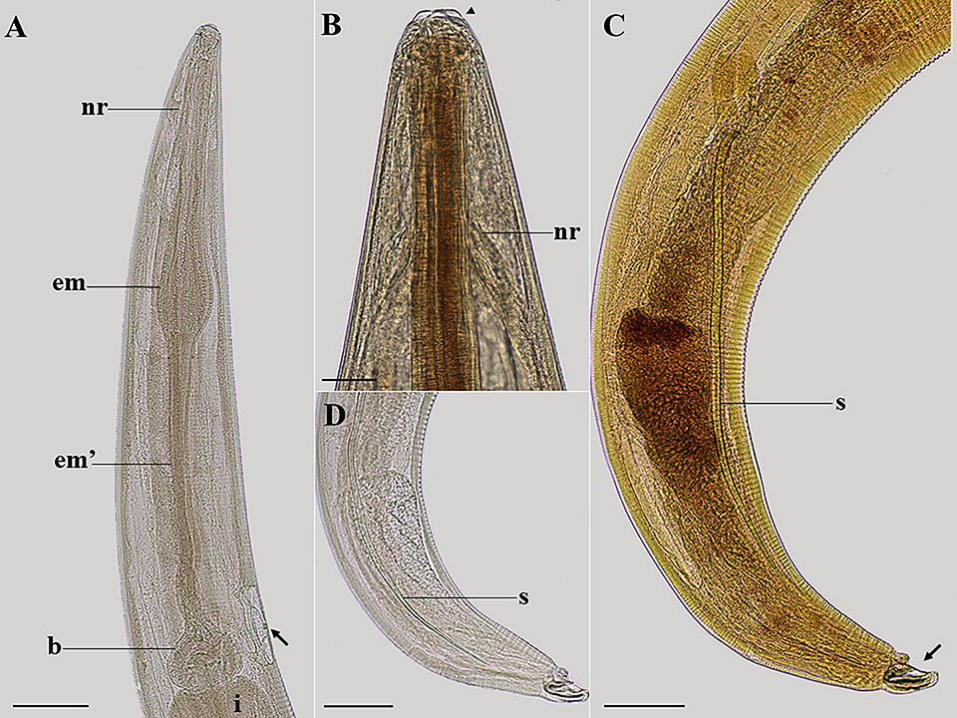
Light microscopy of male *Ozolaimus megatyphlon,* a parasite of *Iguana iguana*. A. Anterior extremity, nerve ring (nr), esophagus first portion (em), and muscular esophagus second portion (em'), prebulbar excretory pore (arrow), bulb (b) and intestine (i). Bar=100µm. B. Anterior extremity, lips (arrowhead) and nerve ring (nr). Bar=50µm. C-D. Posterior end, lateral view, the spicule (s) and tail (arrow). Bar=200µm.

**Figure 4:**
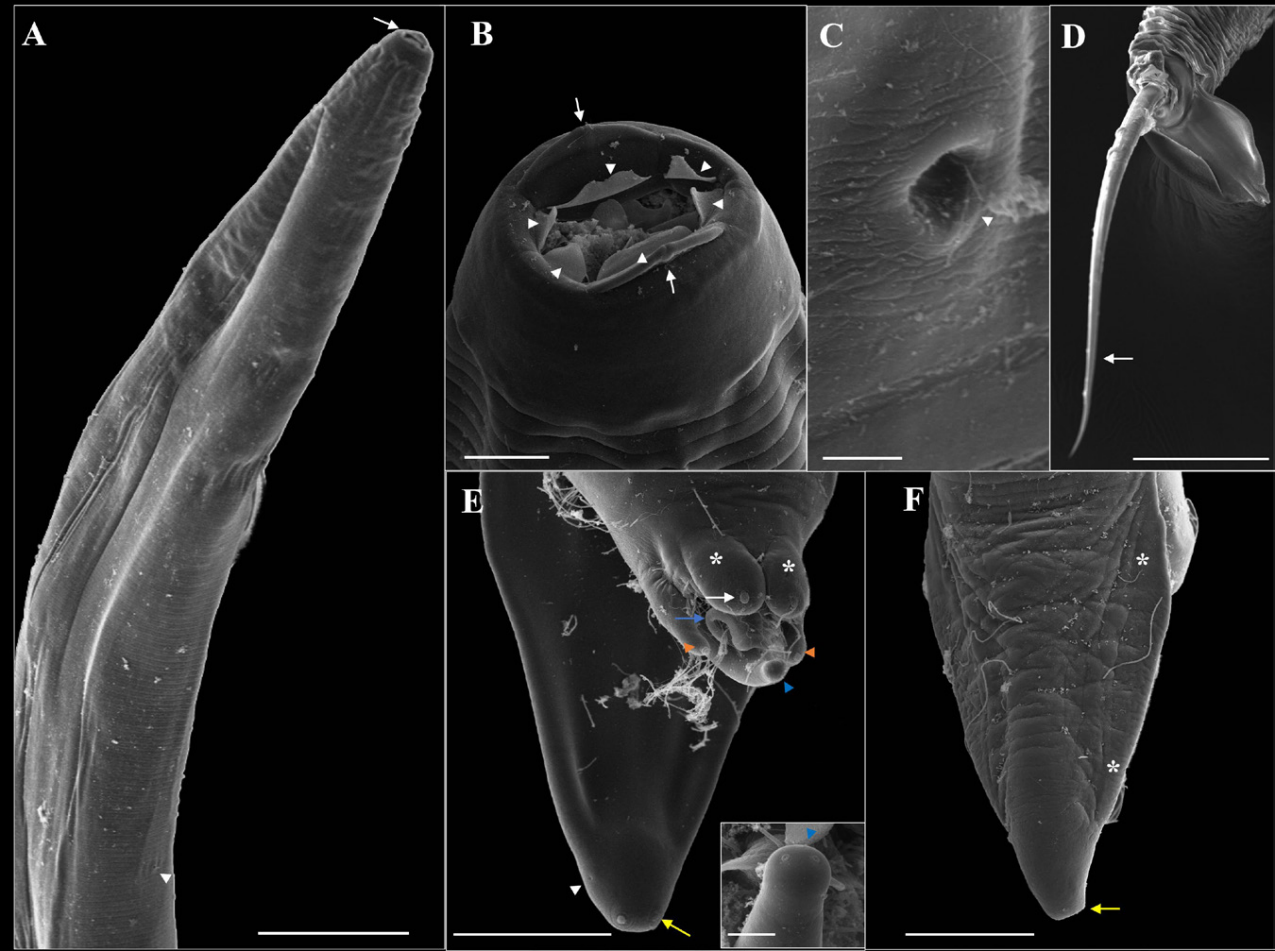
Scanning electron microscopy of male *Ozolaimus megatyphlon,* a parasite of *Iguana iguana*. A. Anterior extremity, observe the cephalic region (arrow) and excretory pore (arrowhead). Bar=500µm. B. Anterior extremity in the cephalic region, one can observe the mouth in a vertical slit delimited by two conspicuous and hemispherical lateral lips containing amphid each (arrow), projection of six cuticular membranes (arrowhead). Bar=20µm. C. Observe the excretory pore (arrowhead). Bar=5µm. D. Posterior end, exposed spicule (arrow) and retracted tail. Bar=100µm. E. Posterior end, lateral and ventral view, two pairs of papillae can be seen: a large pair (*), in a ventral and precloacal position and the other small pair located at the tip of the tail (arrow). Around the cloaca there are two pairs of appendages: a large pair a in the dorsolateral position (orange arrowhead), bar= 50μm, and the other small membranous pair occupying a ventrolateral position, (white arrow) and genital cone (blue arrowhead). Caudal papillae (yellow arrow). Phasmids (white arrowhead). Bar=50μm; Insert genital cone (blue arrowhead). Bar=10μm. F. Caudal wings (*) well developed are present, originating immediately anterior to the tail insertion and ending immediately anterior to the caudal papillae (yellow arrow). Bar=25µm.

Medium-sized parasite, rounded body with cuticle completely striated transversely. Two lateral lips and a triangular oral opening, with small, rounded projections below the esophageal leaves. Esophagus long, thin, almost cylindrical, and divided into two portions: the first comprises the dilatation region, and the second goes to the bulb, the second portion being longer than the first. Bulb well developed; excretory pore immediately anterior or at the level of the bulb; nerve ring near the first portion of the esophagus. Males have a projection of six cuticular membranes from the esophageal segment. Spicule short and pointed. Genital cone present. Tail differentiated, short, and curved, containing a pair of precloacal papillae, a pair of postcloacal papillae, and a pair of caudal papillae. Females with vulva covered by a very prominent vulvar lip and a long uterus. Gravid females have ovoid, thin-shelled eggs and no embryonated.

**Based on 25 male specimens:** body 5.31 mm (4.71-6.28) length and 0.42 (0.33-0.61) wide at the bulb region. Distances from anterior end to nerve ring and excretory pore 0.26 (0.10-0.80) and 1.74 (1.08-2.14), respectively. Elongated esophagus measuring 1.63 (1.20-1.90) in length, divided into two portions where the first portion measures 0.72 (0.50-0.86) in length and 0.14 (0.10-0.16) wide; the second portion measures 0.69 (0.30-1.02) in length and 0.09 (0.04-0.53) wide. Esophageal bulb measuring 0.19 (0.14-0.28) in length and 0.18 (0.14-0.22) in width. A differentiated shape of the end of the spicule: spicule is short and pointed. Spicule 1.28 (0.80-2.10) in length. Tail measuring 140 (70-230) µm in length. Phasmids present.

**Based on 20 female specimens:** body 6.89 mm (4.80-7.57) length and 0.64 (0.13-0.80) wide at bulb region. Distances from anterior end to nerve ring and excretory pore 0.28 (0.22-0.37) and 2.33 (1.30-2.74), respectively. Elongated esophagus measuring 2.18 (1.56-2.77) length, divided into two portions where the first portion measures 0.98 (0.74-1.20) length and 0.17 (0.07-0.24) wide; the second portion measures 0.99 (0.42-1.37) length and 0.09 (0.05-0.19) wide. Esophageal bulb measuring 0.24 (0.17-0.36) in length and 0.21 (0.13-0.29) in width. Distance from vulva to anus and end of tail: 1.35 (1.00-1.94) and 1.69 (1.03-2.50), respectively. Tail measuring 300 (200-510) µm in length. Phasmids present. Thin-shelled eggs measuring 170 µm (100-980) length by 60 µm (50-80) wide.

### *Ozolaimus cirratus* (based on light microscopy and scanning electron microscopy. Figures 5 [Fig gf06] [Fig gf07] to 8; Table 2)

**Figure 5:**
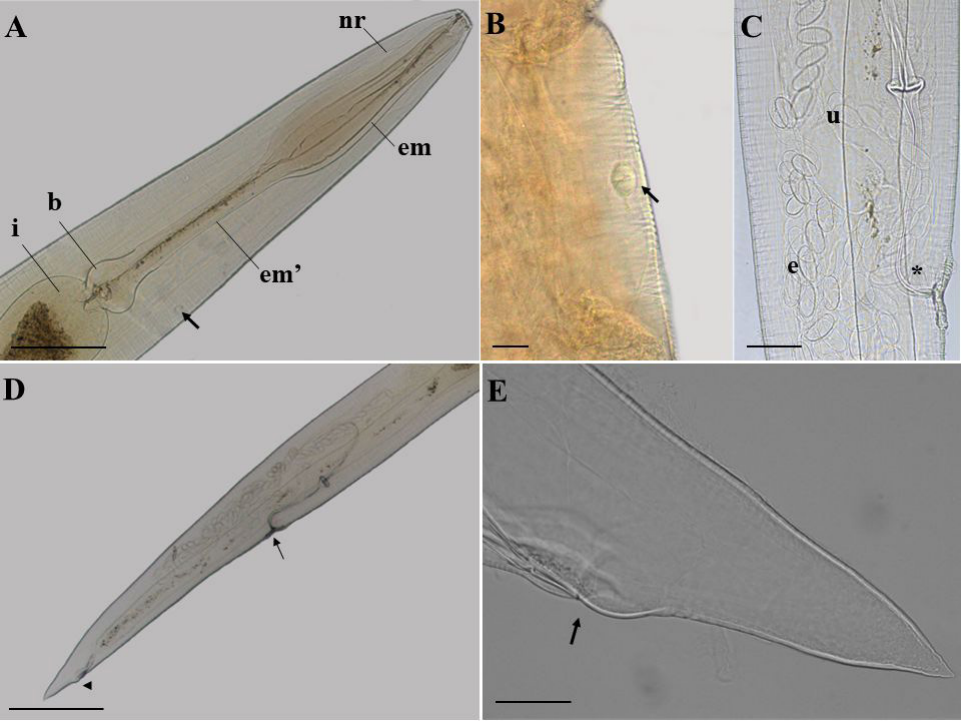
Light microscopy of female *Ozolaimus cirratus,* a parasite of *Iguana iguana*. A. Anterior extremity of the female, lateral view, nervous ring (nr), esophagus first portion (me), and muscular esophagus second portion (me'), prebulbar excretory pore (arrow), bulb (b) and intestine (i). Bar=300 µm. B. Excretory pore, lateral view (arrow). Bar=30µm. C. Vulvar region, note the short, muscular vagina (*), uterus (u) with eggs (e). Bar=100µm. D. Posterior end, lateral view, vulva (arrow) and anal opening (arrowhead). Bar=200µm. E. Posterior end, lateral view, anal opening (arrow). Bar=100µm.

**Figure 6 gf06:**
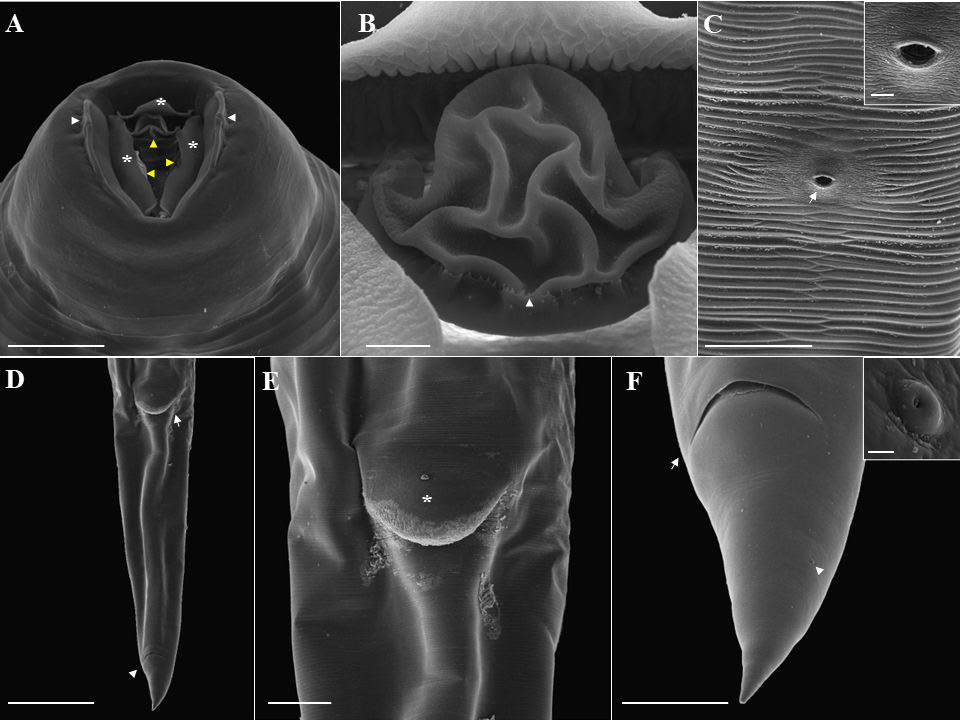
Scanning electron microscopy of female *Ozolaimus cirratus,* a parasite of *Iguana iguana*. A. Anterior end, projection of 3 tripartite cuticular membranes from the esophageal segment (*), amphids (arrowhead), opening of the esophagus into the oral cavity with esophageal projection (yellow arrowhead). Bar=50µm. B. Anterior end, esophageal leaves with serrated structures (arrowhead). Bar=5µm. C. Excretory pore (arrow). Bar= 50µm. Insert excretory pore opening. Bar=5µm. D. Posterior end, ventral view, vulva whose anterior lip projects slightly and protrudes the vulvar opening (arrow), and anal opening (arrowhead). Bar=500µm. E. Posterior end, ventral view, anterior lip of the vulva (*). Bar=100µm. F. Anal opening (arrow), and phasmid (arrowhead). Bar=100µm. Phasmid opening insert. Bar=2µm.

**Figure 7 gf07:**
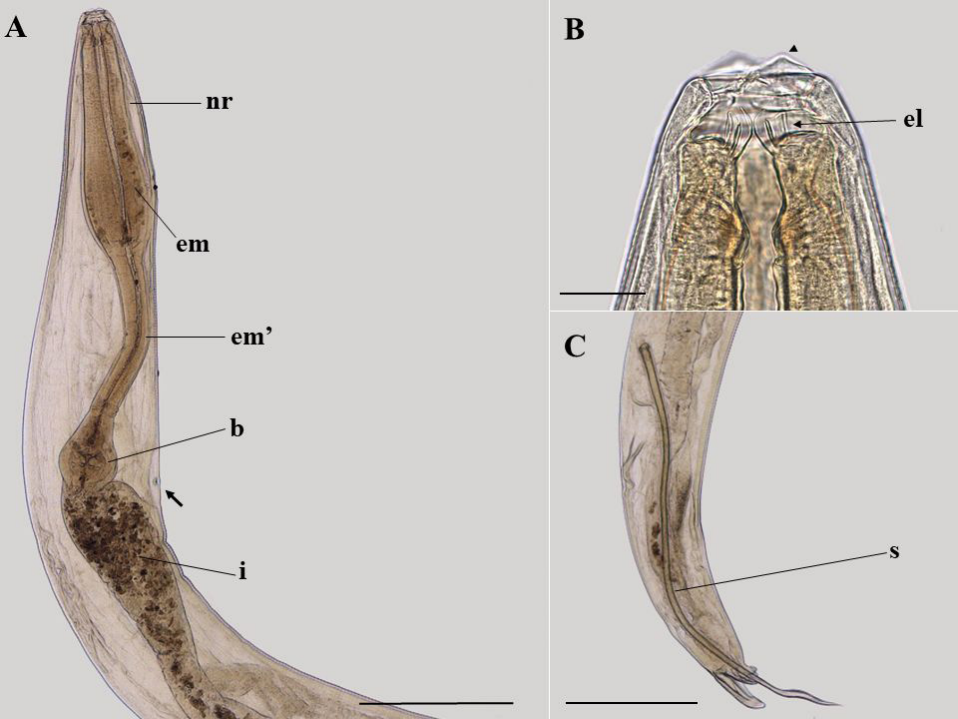
Light microscopy of male *Ozolaimus cirratus,* a parasite of *Iguana iguana*. A. Anterior extremity, nervous ring (nr), esophagus first portion (me), and muscular esophagus second portion (me'), prebulbar excretory pore (arrow), bulb (b) and intestine (i). Bar=500 µm. B. Lips (arrowhead), esophageal leaf (el). C. Posterior end, tail lateral view, spicule (s). Bar=500µm.

**Figure 8:**
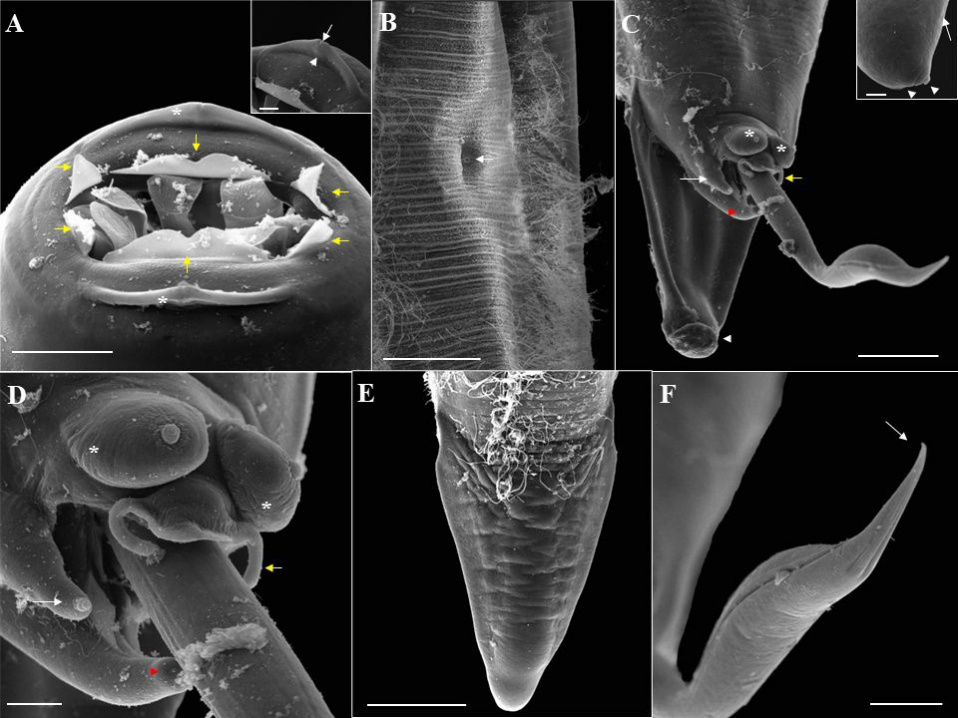
Electron microscopy of male *Ozolaimus cirratus,* a parasite of *Iguana iguana*. A. Anterior extremity in the cephalic region, a vertical slit mouth delimited by two conspicuous and hemispherical lateral lips (*), projection of six cuticular membranes (yellow arrow). Bar=20µm. Insert, lateral lip containing amphid (arrowhead), and orifice of the amphids canaliculus (arrow). Bar=5µm. B. Note the excretory pore (arrow). Bar=100µm. C-D. Posterior end, lateral and ventral view, two pairs of papillae can be observed, and a large pair (*), in a ventral and precloacal position and the other small pair located at the tip of the tail (arrowhead). Around the cloaca there are two pairs of appendages, one pair large and in the dorsolateral position (white arrow) containing a papilla at the end, genital cone containing two papillae (red arrowhead), and the other pair small membranous occupying a ventrolateral position (yellow arrow). Bar= 50µm. Insert caudal papillae (arrowhead) and phasmids (arrow). Bar= 5µm. E. Posterior end, tail dorsal view. Bar=50µm. F. Tip of the spicule. Bar=20µm.

Medium-sized parasite, rounded body with cuticle totally striated transversely. Dorsoventrally elongated buccal capsule with two lateral lips, and a triangular-shaped oral opening, and with serrated projections below the esophageal leaves. Esophagus long and divided into two portions: the first portion comprises the dilatation region, and the second portion is the one that goes to the bulb, the first portion being longer than the second. Bulb well developed; excretory pore immediately anterior or at the level of the bulb; nerve ring near the first portion of the esophagus. Males have a curved tail. Spicule long, distal end of the spicule is curved, and it is pointed. Genital cone present. Females with long uterus and vulva covered by a prominent vulvar lip. Gravid females thin-shelled eggs and no embryonated.

**Based on 25 male specimens:** body 5.95 mm (5.00-7.28) length and 0.49 (0.40-0.63) wide at the bulb region. Distances from anterior end to nerve ring and excretory pore 0.23 (0.17-0.29) and 1.77 (1.19-2.01), respectively. Elongated esophagus measuring 1.50 (1.12-2.15) in length, divided into two portions where the first measures 0.64 (0.52-0.74) in length and 0.17 (0.14-0.20) wide; the second measures 0.57 (0.36-0.72) in length and 0.09 (0.06-0.13) wide. Esophageal bulb measuring 0.20 (0.14-0.27) in length and 0.18 (0.11-0.20) in width. A differentiated shape of the end of the spicule: spicule long, distal end of spicule curved, tapering to a point. Spicule 2.08 (1.50-2.37) in length. Tail measuring 170 µm (110-230) in length. Phasmids present.

**Based on 20 female specimens:** body 6.47 mm (4.46-7.77) length and 0.68 (0.53-0.86) wide at bulb region. Distances from anterior end to nerve ring and excretory pore 0.32 (0.29-0.37) and 2.19 (1.66-3.14), respectively. Elongated esophagus measuring 1.95 (1.55-2.80) in length, divided into two portions where the first portion measures 0.86 (0.71-1.22) in length and 0.22 (0.13-0.31) wide; the second portion measures 0.67 (0.50-1.00) in length and 0.10 (0.04-0.17) wide. Esophageal bulb measuring 0.26 (0.17-0.38) in length and 0.21 (0.17-0.38) in width. Distance from vulva to anus and end of tail: 1.39 (1.05-1.80) and 1.79 (1.42-2.22), respectively. Tail measuring 320 µm (170-510) in length. Phasmids present. Thin-shelled eggs measuring 110 µm (80-120) in length by 60 µm (50-70) in width.

## Discussion

The genus *Ozolaimus* Dujardin, 1845, comprises medium-sized nematodes with a dorsoventrally elongated mouth with two lateral lips and a long esophagus divided into a short anterior portion and a thinner posterior portion ending in a distinct bulb. Side wings absent. Excretory pore at the anterior end; long spicule; short, truncated tail; well developed, curved distally. Females have a vulva anterior to the anus and covered by the vulvar lip, with a long and sinuous uterus (Rudolphi, 1819; Dujardin, 1845; Vicente et al., 1993). In our study, the morphological characters were compatible with this genus.

Five species of *Ozolaimus* parasites on lizards are currently described: *Ozolaimus megatyphlon* was initially described by Rudolphi (1819) found in the caeca of *Iguana iguana* in Berlin; *Ozolaimus cirratus* Linstow, 1906, in the large intestine of *Iguana tuberculata* Laurenti, 1768, in Germany; *Ozolaimus monhystera* (Linstow, 1902) in *Cyclura cornuta* (Bonnaterre, 1789) in Haiti; *Ozolaimus ctenosauri* Caballero, 1938, in the small intestine of *Ctenosaura pectinata* (Wiegmann, 1834) in Mexico; and *Ozolaimus linstowi* Malysheva, 2016 parasitizing the large intestine of *I. iguana* in Mexico. Of these authors, Linstow (1902, 1906) described the drawings referring to the two species *O. cirratus* and *O. megatyphlon* in more morphological detail. This was also the case with Ortlepp (1933), in his drawing of the esophagus and tail of these species. The images and descriptions contributed significantly to differentiating the species in our study. As observed in Table 1 of *O*. *megatyphlon* and Table 2 of *O*. *cirratus*, the morphometry of these species shows little difference between them, and in relation to other works, no significant differences were observed either.

In our research into the morphological analysis by LM, the species *O. cirratus* and *O. megatyphlon* differ from each other by the shape of the esophagus, position of the excretory pore, and shape of the spicule, as observed by Ortlepp (1933). And, for the first time through SEM, the presence of a small, serrated structure in the esophageal leaves of *O. cirratus* was described, while in *O. megatyphlon*, it presented small, spaced, and rounded structures in the esophageal leaves. In addition, phasmids were described for the first time in both species.

Parasites of this genus have been recorded in free-living iguanas in Midwest and Northeast Brazil (Breves et al., 2011; Teles et al., 2017; Otávio et al., 2018). The parasitological indices found in this work differ from those obtained by Teles et al. (2017), who obtained an average of approximately 1.600 *Ozolaimus* sp. per host. These parasites have also been recorded in iguanas in Peru by Arrojo (2002), in Panama by Bursey et al. (2007), and in Colombia and Suriname by Ávila & Silva (2010), demonstrating that these infections are very frequent. In the North region of Brazil, this was the first occurrence in the State of Pará, where 12.028 adult *O*. *megathyphlon* and *O*. *cirratus* were registered with a prevalence of 100% (n = 4), differing from the research by Otávio et al. (2018), which recorded 388 adult pinworms of *O*. *megatyphlon* and *O*. *cirratus* with a prevalence of 60% (n = 5), and Teles et al. (2017), which obtained a prevalence of 66.6% (n = 18), both in Brazil. There are still few records of *Ozolaimus* spp. in iguanids in Brazil.

The nematodes studied here, *O. megathyphlon* and *O. cirratus*, have also been recorded in *Iguana rhinolopha, Iguana tuberculate*, and *I. iguana* (Linstow, 1906; Dosse, 1942; Caballero, 1938; Loukopoulos et al., 2007; Breves et al., 2011; Otávio et al., 2018). Both Jacobson (2007) and Teles et al. (2017) report that the most common genera of *Iguana* parasites are *Alaeuris*, *Ozolaimus*, and *Tachygonetria*, and these pinworms have high host specificity, which is common in lizards, chelonians, and some snakes. The eggs, when ingested by the reptile, hatch in the upper digestive tract, which releases the larvae; when mature, adults migrate to the rectum (Greiner & Mader, 2006). They are usually elongated eggs with a flattened side, and most have a subpolar operculum (Anderson, 2000). The form of infection is mainly fecal-oral (Klingenberg, 2007). They are considered commensal organisms and can help in the digestion of foods of plant origin; however, in cases of massive infections, they can cause obstructions of the gastrointestinal tract, cloacal prolapse, and sometimes a slight local inflammatory reaction (Greiner & Mader, 2006; Klingenberg, 2007; Jacobson, 2007).

The iguanas in this research were free-living and had a high infection rate. According to van Marken Lichtenbelt (1993), free-living herbivorous lizards commonly present high loads of oxyurids, reaching up to 5.000 parasites per lizard. Kehoe et al. (2020) recorded an effective measure for controlling these helminths using oxfendazole to treat oxyurid nematodiasis.

Lent & Freitas (1948) identified a single site of infection in the genus *Ozolaimus* sp., the large intestine. Breves et al. (2011) also recorded infection by this nematode in the large intestine, and in coproparasitological and morphological analysis of adult helminths, Carvalho (2018) recorded *Ozolaimus* in the colon of a green iguana. In the present study, these nematodes were present throughout the large intestine, which corroborates the findings of the researchers mentioned above.

## Conclusion

In this work, we report for the first time the infection by *O*. *megatyphlon* and *O*. *cirratus* in green iguanas in the state of Pará, thus expanding the geographical occurrence of the genus. Under scanning electron microscopy, we added distinguishing morphological characters between the two species, such as the presence of a small, serrated structure below the esophageal leaves in *O*. *cirratus* and small, spaced, and rounded structures below the esophageal leaves in *O*. *megatyphlon*. In addition to showing for the first time the phasmids in both species.
